# Physical exposure during patient transfer and risk of back injury & low-back pain: prospective cohort study

**DOI:** 10.1186/s12891-020-03731-2

**Published:** 2020-10-31

**Authors:** Jonas Vinstrup, Markus D. Jakobsen, Pascal Madeleine, Lars L. Andersen

**Affiliations:** 1 National Research Centre for the Working Environment, Lersø Parkallé 105, 2100 Copenhagen Ø, DK Denmark; 2 Sport Sciences – Performance and Technology, Department of Health Science and Technology, Aalborg University, Aalborg, Denmark

**Keywords:** Patient transfer, Biomechanical load, Low-back pain, Back injury, Healthcare

## Abstract

**Background:**

Work-related musculoskeletal disorders (MSDs) are common among healthcare workers. Because frequent patient transfer has been associated with increased risk of MSDs, we aim to quantify the physical load associated with commonly-used assistive devices and to investigate associations between accumulated physical exposure and risk of MSDs.

**Methods:**

By applying an exposure matrix based on objective measurements of electromyography and trunk flexion on a large (*n* = 1285) prospective cohort, intensity of low-back pain (LBP) and odds of back injury at 1-year follow-up were modelled using linear models and logistic regressions, respectively. The cohort was divided into groups according to physical exposure; i.e. low (1st quartile), moderate (2nd and 3rd quartiles) and high (4th quartile) exposure.

**Results:**

Exposure profiles are provided for 9 groups of assistive devices, with ceiling lifts and intelligent beds eliciting the lowest physical exposure. In the fully-adjusted model, we report differences in LBP intensity at follow-up between the low and moderate exposure groups (*p* = 0.0085). No difference was found between the moderate and high exposure groups (*p* = 0.2967). Likewise, we find no associations between physical exposure and odds of back injury at 1-year follow-up, with a prevalence of 11, 13 and 11% for the three groups, respectively.

**Conclusions:**

Low physical exposure during patient transfer was prospectively associated with lower intensity of LBP. Consistent use of assistive devices associated with low physical exposure, namely ceiling-lifts and intelligent beds, may play a role in reducing the incidence of MSDs among healthcare workers.

## Background

Work-related musculoskeletal disorders (MSDs) are more frequently reported among healthcare workers compared to other professions [1–4], and 37% of Danish healthcare workers report being hindered in their profession because of pain [5]. Low-back pain (LBP) is the most commonly-cited musculoskeletal complaint among this subgroup of the working population, with a 1-year prevalence ranging between 28 and 96% [1, 6, 7]. In addition to the individual burden of LBP [8], the socioeconomic costs – e.g. sickness absence and loss of productivity – are likewise alarmingly high [9–11], making the current situation in the healthcare industry a societal issue with far-reaching implications. The severity of the situation is furthermore highlighted by the current global shortage of nurses; estimated to increase by 2030 [12–15]. Thus, identifying risk factors with the goal of improving the local working environment is vital for the profession.

The need for identification of potential risk factors and preventative interventions is furthermore reflected in the fact that the afore-mentioned high prevalence of work-related MSDs among healthcare personnel goes back several decades [16–18]. Likewise, the notion that a high frequency of manual patient transfers is associated with increased risk of low-back injury cannot be considered a new finding [19, 20].

Because individualized and/or multimodal approaches are inherently difficult to apply to large populations of the workforce, most interventions to date have focused on identifying possible risk factors that apply to the healthcare profession as a whole [21]. Several of these interventions have focused on the negative consequences of high physical loads throughout the workday [22–24]. For example, a recent prospective cohort study reported that an accumulated high volume of physical workload was associated with increased risk of overall poor health [22]. Following this, some of the most promising interventions aiming to reduce the physical load among healthcare workers seem to be the ones focusing on decreasing the frequency and/or intensity of manual lifting [25–29]. This is often and most successfully done by increasing the use of assistive devices during patient transfers, as previous studies have reported associations between frequent use and decreased risk of MSDs [30–32]. However, it is currently unknown whether this effect is mainly due to single/specific assistive devices, or if it is related to consistent use of a combination of various assistive devices. Following this, context-specific information based on biomechanical measurements on the physical risk factors associated with patient transfer and the accompanying benefits of utilizing specific assistive devices when appropriate, is lacking. Thus, quantifying the biomechanical exposure associated with the use of different assistive devices would be highly relevant when investigating the risk of back injury and LBP in this population.

Therefore, by combining technical measurements of muscle activity and trunk inclination during patient transfers with a prospective questionnaire design, we sought to create an exposure-matrix to identify associations between biomechanical load during patient transfer and the odds of back injury and LBP among healthcare workers.

## Methods

In relation to this project we have previously published a protocol describing the technical measurements in detail [33] as well as a descriptive article on the relative biomechanical load associated with the included assistive devices [34]. Therefore, the present article refers to these publications and directs its focus on the methods related to the development of exposure profiles.

### Study design and participants

The survey used in the present study was partially based on the 2018 round of the Danish Work Environment Cohort Study (DWECS) - from which we have previously reported associations between pain, stress and sleep among healthcare workers [35–37] - and included questions concerning lifestyle-, health- and factors related to the work environment. While the entire questionnaire exists only in Danish, the questions used in this study have been translated and included in this section; containing questions specific to the work environment of healthcare workers. The baseline questionnaire was sent out to 3329 healthcare workers during the summer of 2017 with a 1-year follow-up. For the purpose of this analysis, a total of 1285 was included as they fulfilled the criteria corresponding to the population from which the technical measurements were collected: Females working as nurses, nurses´ aides and assistants, physio- or occupational therapists, radiographer or porter, engaging in daily patient transfers including patients who were not completely self-reliant and having experienced no back injury within the previous year and with LBP intensity < 6 (0–10). From this cohort, 710 (55%) responded to the follow-up questionnaire and were included in the analysis. Because both the presence and intensity of LBP are strong predictors of future LBP [38], we included only healthcare workers with low pain levels and who were injury-free in order to determine if low biomechanical load would be associated with a preventative effect against LBP. Table 1 shows baseline demographics, work-, health- and lifestyle variables for all healthcare workers and for the low, moderate and high exposure groups.

**Table 1:** Baseline demographics, work-, health- and lifestyle variables for all healthcare workers and for the low, moderate and high exposure groups

	All			Low			Moderate			High		
Variable	Mean	SD	%	Mean	SD	%	Mean	SD	%	Mean	SD	%
**N**	1285			321			643			321		
**Female**			100			100			100			100
**Age (y)**	46.8	11.3		45.3	11.3		46.3	11.6		49.1	10.3	
**BMI**	24.9	4.6		25.0	4.7		25.2	4.9		24.3	3.6	
**Smokers**			9.0			9.3			8.9			7.8
**Years in profession**	17.8	11.9		16.1	11.0		17.6	12.3		19.6	11.6	
**Working hours/week**	34.7	3.4		34.8	3.3		34.7	3.3		34.7	3.5	
**LBP within the previous 4 weeks (0–10)**	1.5	1.6		1.5	1.6		1.5	1.7		1.4	1.6	
**Back injuries within the previous 12 months**	0			0			0			0		
**Frequency of patient transfers with more than 1 healthcare worker:**												
0/4			3.5			2.9			2.2			6.7
1/4			19.4			12.8			25.2			14.3
2/4			30.8			29.1			35.0			23.9
3/4			26.4			35.8			25.8			18.4
4/4			19.9			19.4			11.9			36.8
**Frequency of patient transfers with patients being so self-reliant that no assistive device is necessary:**												
0/4			17.5			32.9			13.8			9.8
1/4			31.3			30.4			34.7			25.4
2/4			30.9			23.0			34.2			32.1
3/4			20.3			13.7			17.3			32.7
4/4			0			0			0			0
**Level of leisure-time physical activity within the previous 12 months:**												
Sedentary			5.1			4.7			6.1			3.7
Light exercise > 3/week			63.4			60.4			66.1			61.4
Moderate exercise > 3/week			28.3			30.8			25.0			31.5
Vigorous exercise several times per week			3.2			4.1			2.8			3.4

This study utilizes the combination of technical measurements and a prospective questionnaire design. Measurements of bilateral erector spinae electromyographic activity (EMG) and trunk forward- & lateral flexion (actigraphy) during patient transfers throughout an entire workday were acquired from 52 female healthcare workers, using wireless equipment (TeleMyo DTS Telemetry, Noraxon, AZ, USA). EMG values consisted of normalized (% of max), 95th percentile ranks from the merged value of the erector spinae muscles [34].

The results from the technical measurements were used to create an exposure profile for each individual assistive device, comprised of weighted means from EMG- and accelerometer data. “No assistive device” was used as reference and given the value “1”. All other assistive devices were assigned exposure profiles relative to this value, based on their combined values from EMG- and accelerometer data in the following manner: The normalized values were divided by the reference to achieve a fraction (e.g. nRMS ceiling-lift/“no assistive device”; 24.0/27.9 = 0.86), and the average of the EMG- and accelerometer values was calculated. In order to weigh the contribution from EMG and kinematics more equally and hereby emphasize the former [39], the average of the two kinetic values was used to calculate the exposure profile; i.e. the average of forward- and lateral flexion represented a flexion value (e.g. 22.3/36.5 = 0.61 and 24.8/32.1 = 0.77 for forward- and lateral flexion, respectively; averaging 0.69), which was then used to calculate the average of the combined EMG- and accelerometer values (e.g. nRMS (0.86) and trunk flexion (0.69) = 0.77 for the ceiling-lift). The normalized mean values utilized in creating the present exposure matrix are found in the descriptive article related to this project, in which descriptions of the included assistive devices as well as demographics of the participants who partook in the field measurements, are found [34]. In short, the measurements entailed 14 different assistive devices which were subsequently grouped according to function; e.g. the wheelchair and walking-rollator were characterized as “walking aids” whereas the turner transfer and stand-assist were characterized as “standing aids”. Additionally, the ceiling-lift and accompanying sling were regarded as one, resulting in a total of 9 groups of assistive devices (Table 2).

**Table 2:** Exposure profiles for assistive devices

Assistive device	Index	EMG	Forward flexion	Lateral flexion
No assistive device	**1**	1	1	1
Hospital bed	**0.8600**	0.9211	0.5492	1.0486
Intelligent bed	**0.8246**	0.8566	0.6792	0.9060
Bed sheet	**1.0289**	1.0968	1.0065	0.9155
Walking aids	**1.0200**	0.9892	1.0440	1.0573
Masterturner	**0.8582**	0.9606	0.7903	0.7215
Sliding sheet	**1.0109**	1.0860	1.0455	0.8259
Ceiling-lift	**0.7762**	0.8602	0.6123	0.7721
Sliding board	**1.0264**	1.2007	1.0788	0.6253
Standing aids	**0.8517**	0.9283	0.8372	0.7130

Exposure profiles based on the weighted contribution of EMG, forward- and lateral flexion values obtained during full-day field measurements of patient transfers performed in hospitals [34]

By using the exposure profile of each assistive device (technical measurements) and the quantification of frequency of use (survey information), each participant was assigned individual exposure values. Therefore, the individual exposure values were created by initially identifying the relative EMG, forward- and lateral flexion values (i.e. exposure value) inherent to each assistive device, multiplied with the frequency of use for each participant. That is, the more frequent the participant used an assistive device with a certain profile, the closer the participant’s exposure value would be to that of the specific assistive device. The participants were then grouped into quartiles based on their exposure, and the two middle quartiles (25–75%) were grouped to represent the norm. Following this, we tested associations between low exposure (1st quartile; *n* = 175), moderate (2nd and 3rd quartiles, *n* = 349) and high (4th quartile, *n* = 186) and the outcomes described in the following.

### Outcome variables

Back injury and LBP at follow-up were assessed by the following survey questions:

1. *Rate your average pain for the low back within the previous 4 weeks (0–10).*
2. *Have you injured your back during patient transfer within the previous 12 months? (yes/no) (Recall if the accident happened suddenly and unexpected)*

### Control variables

We include 9 groupings of assistive devices (Table 2), all of which included in the questionnaire survey. The quantitative use of each individual assistive device was assessed with the following question, with five possible response options ranging from 0/4 (almost never) to 4/4 (every time): “*How often do you use this assistive device during patient transfer?”*

Frequency of patient transfers was evaluated with the question:

*“How many patients do you transfer per day?”* with possible responses ranging from 1) none, 2) less than one per day (e.g. 2–3 per week), 3) 1–2 per day, 4) 3–4 per day, 5) 5–6 per day, 6) 7–8 per day. 7) 9–10 per day to 8) more than 10 per day.

Frequency of patient transfers performed together with one or more colleagues was evaluated with the question: *“How often are you more than one care worker to do the transfer?”* with five possible response options ranging from 0/4 (almost never) to 4/4 (every time).

Finally, self-reliance of the patients was evaluated by asking “*How many of your patients are so self-reliant that it is not necessary to use assistive devices during transfers?*“, again with five response options again ranging from 0/4 (virtually none) to 4/4 (all patients).

### Covariates

In the results section we report fully-adjusted associations between individual exposure values and the outcomes of back injury and low-back pain. The analyses control for the following possible confounders relating to the individual, psychosocial- and work environment as well as to the patient transfer scenario itself:

Age, body mass index, smoking, education, physical activity during leisure time, pain intensity < 6 and no back injury within the previous 12 months at baseline, seniority, working hours, overall mental health as well as work-related attitudes towards justice, teamwork, influence, emotional demands, clarity of tasks as well as management recognition and support. For example, the latter was evaluated by the questions “*do you feel your work is recognized and appreciated by management*?” and “*do you get the help and support you need from management?*”

Furthermore, we also adjusted for frequency and number of personnel participating in the patient transfer as well as patient self-reliance.

### Statistics

All associations were modelled using the General Linear Mixed Model of SAS version 9.4, which can be used for both logistic regression and linear models. Back injury was modelled as a binary outcome (yes/no) during 1-year follow-up, i.e. logistic regression. LBP intensity was modelled as a continuous outcome at 1-year follow-up, i.e. a linear model. Both analyses were controlled for the covariates mentioned previously, and results are reported as odds ratio (OR) and least square mean differences, respectively, for the lower and upper quartiles in relation to the two middle quartiles (reference). All estimates are provided with 95% confidence intervals and corresponding *P*-values, with the significance level set to *P* < 0.05.

## Results

We report exposure profiles for 9 groups of assistive devices, showing that ceiling lifts, intelligent beds, standing aids, masterturners, and hospital beds - in ascending order - all elicit low exposure relative to “no assistive device”. Contrastingly, assistive devices characterized by a more manual approach to patient transfer (e.g. bed sheet, sliding sheet and sliding board) exhibit higher values of physical exposure (Table 2). Additionally, the incidence of back injuries at follow-up were similar between groups; i.e. 11, 13 and 11%, for the low, moderate and high exposure groups, respectively.

The odds of LBP and back injury at follow-up between groups are shown in Tables 3 and 4, presented as odds ratios (OR) and based on the minimally- and fully-adjusted models, respectively: With the moderate exposure group as reference, we find no significant differences in OR when comparing the low- and high exposure groups (*p* > 0.05). In contrast, we report a significant difference in low-back pain at follow-up between the low and moderate exposure groups (− 0.47, *p* = 0.0219 and − 0.50, *p* = 0.0085 in the minimally- and fully-adjusted models, respectively). Lastly, no difference was found between the moderate- and high exposure groups (*p* > 0.05).

**Table 3:** Physical exposure and odds of back injury & LBP at follow-up. Values are based on the minimally-adjusted model and presented as odds ratios (OR) and differences between least square means (LSM), respectively. CI; confidence intervals

	Back Injury		LBP	
Exposure	OR (95% CI)	***p***-value	LSM (95% CI)	***p***-value
2nd & 3rd quartiles (comparator)	1		1.93	
1st quartile	1.28 (0.63–2.57)	0.4970	− 0.47 (− 0.87 - (− 0.06))	0.0219
4rd quartile	1.32 (0.66–2.66)	0.4295	− 0.24 (− 0.63–0.16)	0.2379

Adjusted for age and frequency of patient transfer

**Table 4:** Physical exposure and odds of back injury & LBP at follow-up. Values are based on the fully-adjusted model and presented as odds ratios (OR) and differences between least square means (LSM), respectively. CI; confidence intervals

	Back Injury		LBP	
Exposure	OR (95% CI)	***p***-value	LSM (95% CI)	***p***-value
2nd & 3rd quartiles (comparator)	1		1.81	
1st quartile	1.14 (0.52–2.51)	0.7367	− 0.50 (− 0.89 - (− 0.13))	0.0085
4rd quartile	1.36 (0.63–2.92)	0.4309	− 0.19 (− 0.57–0.18)	0.2967

Adjusted for age, body mass index, smoking, education, physical activity, LBP, back injury, frequency of patient transfer and number of participating personnel, patient self-reliance, seniority, working hours, overall mental health and work-related attitudes towards justice, teamwork, influence, emotional demands, clarity of tasks as well as management recognition and support

## Discussion

The main finding of this study is that low levels of physical exposure during patient transfer are associated with lower odds of LBP at 1-year follow-up, whereas no associations were found between exposure and the outcome of back injury.

Additionally, we provide exposure profiles for the most commonly utilized assistive devices and show that use of the more comprehensive systems, e.g. ceiling-lifts and intelligent beds, generally result in low biomechanical load during patient transfers.

### Risk of back injury and LBP

Regarding the odds of sustaining a back injury due to accumulated high workload, our results are somewhat in contrast to a number of studies showing decreased injury rates with the implementation of various lifting policies designed to limit manual handling [25, 27, 28, 40–43]. Several of these studies investigate and credit the ceiling-lift [25, 27, 44–46], which we presently show to elicit the lowest biomechanical load among the included assistive devices. However, these results contrast the findings of recent systematic reviews; showing that manual handling training by itself does not lower the risk of musculoskeletal injuries [21, 47]. Therefore, assuming that (increased) use of appropriate assistive devices constitutes the main implementation-strategy of manual handling training, it is unlikely that any effect of increasing the use of assistive devices is due solely to a decrease in physical workload. In this study, this notion is further supported by the finding that the high exposure group did not experience increased odds of adverse outcomes at follow-up. Although somewhat counter-intuitive when viewed through a biomechanical lens, this finding gives thought to the hypothesis that individuals with relatively high levels of physical capacity - through experience - share a less catastrophizing view on manual lifting and may indeed benefit from a progressively increasing workload [48, 49].

In the case of LBP, our finding that low biomechanical exposure during patient transfers is associated with a decrease in pain intensity, is adding to an already confused body of research: As is the case with the outcome of back injury, there is presently no convincing evidence of efficacy for any single intervention preventing LBP in workers [21, 50–52]. Despite this apparent conundrum, appropriate use of assistive devices during patient transfers has been associated with decreased risk of MSDs [26, 31]. However, several distinct work-related factors, including but not limited to work pace, night shifts, standing work, sitting work, static postures, emotional demands, social relations at work, frequent low mood, job strain- and dissatisfaction etc., have also been shown to influence the risk of MSDs [11, 31, 53–56]. In fact, nurses themselves attribute more than 50% of their work-related injuries to inadequate instruction and staffing [57]; illustrating the prevailing attitudes and beliefs among healthcare workers.

Based on the apparent controversy - likely originating from low-quality studies aiming to identify one intervention to rule them all - it is evident that multiple factors contribute to the high prevalence of MSDs in this population of the workforce [31, 53, 54, 58]. With the present analysis we add to the literature by showing that - even when accounting for several known confounders - consistent and appropriate use of technologically-advanced assistive devices may provide a small protective effect against increases LBP among healthcare workers. Within the complex model of the biopsychosocial approach to health and its growing role within healthcare [59, 60], it is therefore not unlikely that the “bio”-aspect can be improved upon by diminishing the accumulative physical load throughout a day, month and career [22–24].

### Perspectives

We have previously reported that healthcare workers in Danish hospitals utilize assistive devices during less than half of patient transfers [34]. It may seem counter-intuitive of a working population characterized by experiencing a high prevalence of MSDs and end-of-day fatigue, to perform the majority of patient transfers without the use of assistive devices. However, because the most commonly-reported barriers for appropriate use of assistive devices include time-restraints and equipment availability [61–63], it is likely that these factors modulate and indeed question which assistive device, if any, is appropriate in the multi-faceted situation that constitutes the patient transfer scenario. Following this, a recent prospective study by Kucera and colleagues investigated multiple factors associated with appropriate use of assistive devices, and found that – in addition to the frequently reported staff- and equipment availability [64] – patient characteristics such as medical condition, mobility level and the presence of physical- or mental impairments were associated with the use of assistive devices [62]. Likewise, a 2019- Cochrane Review indicates that patients influence the healthcare personnel’s practice and performance in numerous ways [65], and several studies illustrate how a myriad of work-related factors (e.g. self-efficacy, organizational safety climate, adequate guidance, job strain – and dissatisfaction, time-restraints, easiness of use, equipment location and compatibility, patient preference etc.) influence whether or not healthcare workers engage in the use of appropriate assistive devices [54, 57, 62, 63, 66].

Collectively, the overall message is therefore that a broad array of situational-specific factors contributes to the decisions made by healthcare workers during patient transfer. As indicated by the ever-increasing number of largely ineffective single-mode interventions, it seems evident that the multi-factorial issue at hand is hardly solved by one type of intervention alone [50, 66–69].

### Strengths and limitations

Limitations of this study primarily include the inherent recall- and non-response bias that accompanies prospective questionnaire designs [70], potentially resulting in overrepresentation of individuals more conscious of their health [71]. Additionally, the innate uncertainty when reporting subjective outcomes such as injury and pain intensity should not be overlooked [72]. Regarding drop-out, the response-rate at follow-up is well within what is considered normal for questionnaire surveys [73, 74], and differences between responders and non-responders have been elucidated previously [30]. The potential issue regarding generalizing physical exposure associated with specific assistive devices based on technical measurements on a subgroup of healthcare workers as well as the inherent limitations of using EMG- and kinematic measurements as indicators of biomechanical exposure, have also been discussed previously [34].

Strengths of this study include the combination of technical measurements with a prospective design, which allows for applying objectively measured indicators of biomechanical exposure to a large cohort of healthcare workers. Furthermore, the exposure matrix presented for commonly-used assistive devices is believed to prove highly useful in everyday practical settings, as it provides a level of detail novel to the field.

## Conclusion

Low physical exposure during patient transfer is prospectively associated with lower odds of LBP at follow-up. Consistent use of assistive devices associated with lower exposure, e.g. ceiling-lifts and intelligent beds, may be important in reducing the high prevalence of MSDs among healthcare workers. Hospitals aiming to improve the local work environment could therefore benefit from further implementing specific assistive devices.

## Data Availability

Researchers interested in the data should contact the project leader Lars L. Andersen.

## Abbreviations

LBP: Low-back pain
MSD: Musculoskeletal disorder
EMG: Electromyography
nRMS: Normalized root mean square
OR: Odds ratio

## References

[1] Anderson SP, Oakman J (2016). Allied health professionals and work-related musculoskeletal disorders: a systematic review. Saf Health Work.

[2] Cohen-Mansfield J, Culpepper WJ, Carter P (1996). Nursing staff Back injuries: prevalence and costs in long term care facilities. AAOHN J.

[3] Guo HR, Tanaka S, Cameron LL, Seligman PJ, Behrens VJ, Ger J (1995). Back pain among workers in the United States: national estimates and workers at high risk. Am J Ind Med.

[4] Oranye NO, Bennett J (2018). Prevalence of work-related musculoskeletal and non-musculoskeletal injuries in health care workers: the implications for work disability management. Ergonomics..

[5] National Research Centre for the Working Environment (2018). Arbejdsmiljø og Helbred i Danmark 2018.

[6] Bos E, Krol B, van der Star L, Groothoff J (2007). Risk factors and musculoskeletal complaints in non-specialized nurses, IC nurses, operation room nurses, and X-ray technologists. Int Arch Occup Environ Health.

[7] Davis KG, Kotowski SE (2015). Prevalence of musculoskeletal disorders for nurses in hospitals, long-term care facilities, and home health care: a comprehensive review. Hum Factors.

[8] Seidler AL, Rethberg C, Schmitt J, Nienhaus A, Seidler A (2017). Health utilities for chronic low back pain. J Occup Med Toxicol.

[9] Andersen LL, Clausen T, Persson R, Holtermann A (2012). Dose-response relation between perceived physical exertion during healthcare work and risk of long-term sickness absence. Scand J Work Environ Health.

[10] Hansson EK, Hansson TH (2005). The costs for persons sick-listed more than one month because of low back or neck problems. A two-year prospective study of Swedish patients. Eur Spine J.

[11] Ribeiro T, Serranheira F, Loureiro H (2017). Work related musculoskeletal disorders in primary health care nurses. Appl Nurs Res.

[12] Auerbach DI, Buerhaus PI, Staiger DO (2017). How fast will the registered nurse workforce grow through 2030? Projections in nine regions of the country. Nurs Outlook.

[13] Buchan J, Aiken L (2008). Solving nursing shortages: a common priority. J Clin Nurs.

[14] Juraschek SP, Zhang X, Ranganathan V, Lin VW (2012). United States registered nurse workforce report card and shortage forecast. Am J Med Qual.

[15] Zhang X, Tai D, Pforsich H, Lin VW (2018). United States registered nurse workforce report card and shortage forecast: a revisit. Am J Med Qual.

[16] Jensen RC (1987). Disabling back injuries among nursing personnel: research needs justification. Res Nurs Health.

[17] Magora A (1970). Investigation of the relation between low back pain and occupation. IMS Ind Med Surg.

[18] Stubbs DA, Buckle PW, Hudson MP, Rivers PM, Worringham CJ (1983). Back pain in the nursing profession. I. Epidemiology and pilot methodology. Ergonomics..

[19] Owen BD (1989). The magnitude of low-back problem in nursing. West J Nurs Res.

[20] Stobbe TJ, Plummer RW, Jensen RC, Attfield MD (1988). Incidence of low back injuries among nursing personnel as a function of patient lifting frequency. J Saf Res.

[21] Richardson A, McNoe B, Derrett S, Harcombe H (2018). Interventions to prevent and reduce the impact of musculoskeletal injuries among nurses: a systematic review. Int J Nurs Stud.

[22] Andersen LL, Fallentin N, Thorsen SV, Holtermann A (2016). Physical workload and risk of long-term sickness absence in the general working population and among blue-collar workers: prospective cohort study with register follow-up. Occup Environ Med.

[23] Møller A, Mänty M, Andersen LL, Siersma V, Lund R, Mortensen OS (2019). Cumulative physical workload and mobility limitations in middle-aged men and women: a population-based study with retrospective assessment of workload. Int Arch Occup Environ Health.

[24] Oakman J, de Wind A, van den Heuvel SG, van der Beek AJ. Work characteristics predict the development of multi-site musculoskeletal pain. Int Arch Occup Environ Health. 2017;90(7):653–61. 10.1007/s00420-017-1228-9. Epub 2017 May 9. PMID: 28488112. 10.1007/s00420-017-1228-9 28488112

[25] Alamgir H, Yu S, Fast C, Hennessy S, Kidd C, Yassi A (2008). Efficiency of overhead ceiling lifts in reducing musculoskeletal injury among carers working in long-term care institutions. Injury..

[26] Andersen LL, Burdorf A, Fallentin N, Persson R, Jakobsen MD, Mortensen OS (2014). Patient transfers and assistive devices: prospective cohort study on the risk for occupational back injury among healthcare workers. Scand J Work Environ Health.

[27] Chhokar R, Engst C, Miller A, Robinson D, Tate RB, Yassi A (2005). The three-year economic benefits of a ceiling lift intervention aimed to reduce healthcare worker injuries. Appl Ergon.

[28] Collins JW, Wolf L, Bell J, Evanoff B (2004). An evaluation of a “best practices” musculoskeletal injury prevention program in nursing homes. Injury Prev.

[29] Koppelaar E, Knibbe HJJ, Miedema HS, Burdorf A (2012). The influence of ergonomic devices on mechanical load during patient handling activities in nursing homes. Ann Occup Hyg.

[30] Andersen LL, Vinstrup J, Villadsen E, Jay K, Jakobsen MD. Physical and Psychosocial Work Environmental Risk Factors for Back Injury among Healthcare Workers: Prospective Cohort Study. Int J Environ Res Public Health. 2019;16(22):4528. 10.3390/ijerph16224528. PMID: 31731806; PMCID: PMC6887976. 10.3390/ijerph16224528 PMC6887976 31731806

[31] Boocock MG, Trevelyan F, Ashby L, Ang A, Diep N, Teo S, Bagnara S, Tartaglia R, Albolino S, Alexander T, Fujita Y (2019). The influence of psychosocial and patient handling factors on the musculoskeletal health of nurses. Proceedings of the 20th congress of the international ergonomics association (IEA 2018).

[32] Holtermann A, Clausen T, Jørgensen MB, Aust B, Mortensen OS, Burdorf A (2015). Does rare use of assistive devices during patient handling increase the risk of low back pain? A prospective cohort study among female healthcare workers. Int Arch Occup Environ Health.

[33] Vinstrup J, Madeleine P, Jakobsen MD, Jay K, Andersen LL (2017). Patient transfers and risk of Back injury: protocol for a prospective cohort study with technical measurements of exposure. JMIR Res Protoc.

[34] Vinstrup J, Jakobsen MD, Madeleine P, Andersen LL. Biomechanical load during patient transfer with assistive devices: Cross-sectional study. Ergonomics. 2020;63(9):1164–174. 10.1080/00140139.2020.1764113. Epub 2020 May 21. PMID: 32362200. 10.1080/00140139.2020.1764113 32362200

[35] Vinstrup J, Jakobsen MD, Andersen LL (2020). Poor sleep is a risk factor for low-Back pain among healthcare workers: prospective cohort study. Int J Environ Res Public Health.

[36] Vinstrup J, Jakobsen MD, Calatayud J, Jay K, Andersen LL. Association of Stress and Musculoskeletal Pain with Poor Sleep: cross-sectional study among 3,600 hospital workers. Front Neurol. 2018;9. 10.3389/fneur.2018.00968. 10.3389/fneur.2018.00968 PMC6258880 30519210

[37] Vinstrup J, Jakobsen MD, Andersen LL (2020). Perceived stress and low-Back pain among healthcare workers: a multi-center prospective cohort study. Front Public Health.

[38] Eriksen W (2004). Work factors as predictors of intense or disabling low back pain; a prospective study of nurses’ aides. Occup Environ Med.

[39] Wells R, Van Eerd D, Hägg G (2004). Mechanical exposure concepts using force as the agent. Scand J Work Environ Health.

[40] Engst C, Chhokar R, Miller A, Tate RB, Yassi A (2005). Effectiveness of overhead lifting devices in reducing the risk of injury to care staff in extended care facilities. Ergonomics..

[41] Evanoff B, Wolf L, Aton E, Canos J, Collins J (2003). Reduction in injury rates in nursing personnel through introduction of mechanical lifts in the workplace. Am J Ind Med.

[42] Hunter B, Branson M, Davenport D (2010). Saving costs, saving health care providers’ backs, and creating a safe patient environment. Nurs Econ.

[43] Schoenfisch AL, Lipscomb HJ, Pompeii LA, Myers DJ, Dement JM (2013). Musculoskeletal injuries among hospital patient care staff before and after implementation of patient lift and transfer equipment. Scand J Work Environ Health.

[44] Keir PJ, MacDonell CW (2004). Muscle activity during patient transfers: a preliminary study on the influence of lift assists and experience. Ergonomics..

[45] Marras WS, Knapik GG, Ferguson S (2009). Lumbar spine forces during manoeuvring of ceiling-based and floor-based patient transfer devices. Ergonomics..

[46] Silverwood S, Haddock M (2006). Reduction of musculoskeletal injuries in intensive care nurses using ceiling-mounted patient lifts. Dynamics..

[47] Freiberg A, Euler U, Girbig M, Nienhaus A, Freitag S, Seidler A (2016). Does the use of small aids during patient handling activities lead to a decreased occurrence of musculoskeletal complaints and diseases? A systematic review. Int Arch Occup Environ Health.

[48] Bigos SJ, Holland J, Holland C, Webster JS, Battie M, Malmgren JA (2009). High-quality controlled trials on preventing episodes of back problems: systematic literature review in working-age adults. Spine J.

[49] Setchell J, Costa N, Ferreira M, Makovey J, Nielsen M, Hodges PW (2017). Individuals’ explanations for their persistent or recurrent low back pain: a cross-sectional survey. BMC Musculoskelet Disord.

[50] Van Hoof W, O’Sullivan K, O’Keeffe M, Verschueren S, O’Sullivan P, Dankaerts W (2018). The efficacy of interventions for low back pain in nurses: a systematic review. Int J Nurs Stud.

[51] Verbeek JH, Martimo K-P, Karppinen J, Kuijer PPF, Viikari-Juntura E, Takala E-P. Manual material handling advice and assistive devices for preventing and treating back pain in workers. Cochrane Database Syst Rev. 2011. 10.1002/14651858.CD005958.pub3. 10.1002/14651858.CD005958.pub3 PMC13501096 21678349

[52] Sundstrup E, Seeberg KGV, Bengtsen E, Andersen LL. A systematic review of workplace interventions to rehabilitate musculoskeletal disorders among employees with physical demanding work. J Occup Rehabil. 2020. 10.1007/s10926-020-09879-x. 10.1007/s10926-020-09879-x PMC7716934 32219688

[53] Carneiro P, Braga AC, Barroso M (2017). Work-related musculoskeletal disorders in home care nurses: study of the main risk factors. Int J Ind Ergon.

[54] Lee S-J, Lee JH (2017). Safe patient handling behaviors and lift use among hospital nurses: a cross-sectional study. Int J Nurs Stud.

[55] Sherehiy B, Karwowski W, Marek T (2004). Relationship between risk factors and musculoskeletal disorders in the nursing profession: a systematic review. Occup Ergon.

[56] Vedaa Ø, Harris A, Erevik EK, Waage S, Bjorvatn B, Sivertsen B (2019). Short rest between shifts (quick returns) and night work is associated with work-related accidents. Int Arch Occup Environ Health.

[57] Retsas A, Pinikahana J (2000). Manual handling activities and injuries among nurses: an Australian hospital study. J Adv Nurs.

[58] Stimpfel AW, Brewer CS, Kovner CT (2015). Scheduling and shift work characteristics associated with risk for occupational injury in newly licensed registered nurses: an observational study. Int J Nurs Stud.

[59] Brodal P (2017). A neurobiologist’s attempt to understand persistent pain. Scand J Pain.

[60] Kusnanto H, Agustian D, Hilmanto D (2018). Biopsychosocial model of illnesses in primary care: a hermeneutic literature review. J Family Med Prim Care.

[61] D’Arcy LP, Sasai Y, Stearns SC (2012). Do assistive devices, training, and workload affect injury incidence? Prevention efforts by nursing homes and back injuries among nursing assistants: prevention efforts by nursing homes and back injuries among nursing assistants. J Adv Nurs.

[62] Kucera KL, Schoenfisch AL, McIlvaine J, Becherer L, James T, Yeung Y-L (2019). Factors associated with lift equipment use during patient lifts and transfers by hospital nurses and nursing care assistants: a prospective observational cohort study. Int J Nurs Stud.

[63] Lee S-J, Faucett J, Gillen M, Krause N (2013). Musculoskeletal pain among critical-care nurses by availability and use of patient lifting equipment: an analysis of cross-sectional survey data. Int J Nurs Stud.

[64] Vendittelli D, Penprase B, Pittiglio L (2016). Musculoskeletal injury prevention for new nurses. Workplace Health Saf.

[65] Gyi AA. Patient-mediated interventions to improve professional practice: a Cochrane review summary. Int J Nurs Stud. 2019. 10.1016/j.ijnurstu.2019.03.010. 10.1016/j.ijnurstu.2019.03.010 30940351

[66] Jakobsen MD, Aust B, Kines P, Madeleine P, Andersen LL (2019). Participatory organizational intervention for improved use of assistive devices in patient transfer: a single-blinded cluster randomized controlled trial. Scand J Work Environ Health.

[67] Dawson AP, McLennan SN, Schiller SD, Jull GA, Hodges PW, Stewart S (2007). Interventions to prevent back pain and back injury in nurses: a systematic review. Occup Environ Med.

[68] Goldgruber J, Ahrens D (2010). Effectiveness of workplace health promotion and primary prevention interventions: a review. J Public Health.

[69] Thomas DR, Thomas YLN (2014). Interventions to reduce injuries when transferring patients: a critical appraisal of reviews and a realist synthesis. Int J Nurs Stud.

[70] Choi BCK, Pak AWP. A catalog of biases in questionnaires. Prev Chronic Dis. 2004;2 https://www.ncbi.nlm.nih.gov/pmc/articles/PMC1323316/. Accessed 15 Aug 2019. PMC1323316 15670466

[71] Cheung KL, ten Klooster PM, Smit C, de Vries H, Pieterse ME (2017). The impact of non-response bias due to sampling in public health studies: a comparison of voluntary versus mandatory recruitment in a Dutch national survey on adolescent health. BMC Public Health.

[72] Menzel NN (2008). Underreporting of musculoskeletal disorders among health care workers: research needs. AAOHN J.

[73] Millar MM, Dillman DA (2011). Improving response to web and mixed-mode surveys. Public Opinion Quarterly.

[74] Baruch Y (1999). Response rate in academic studies-a comparative analysis. Hum Relat.

